# Supplementary material for: Identification of photoperiod-induced specific miRNAs in the adrenal glands of Sunite sheep (Ovis aries)
Source: Front Vet Sci. 2022 Jul 22;9:888207. doi: 10.3389/fvets.2022.888207 (PMC9354845; doi:10.3389/fvets.2022.888207)
Supplement: Supplementary file 1 [file Data_Sheet_1.ZIP › Supplementary file/miRDeep2 file.zip!/miRDeep2 file/Know_Structure/LP42A1/oar-mir-323c.pdf]

miRBase precursor : oar-mir-323c
Total read count : 133
oar-miR-323c read count : 132
remaining reads : 1

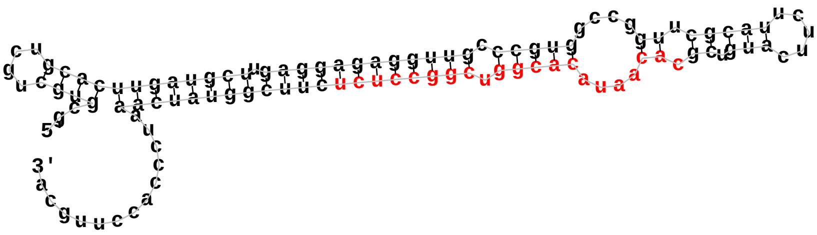

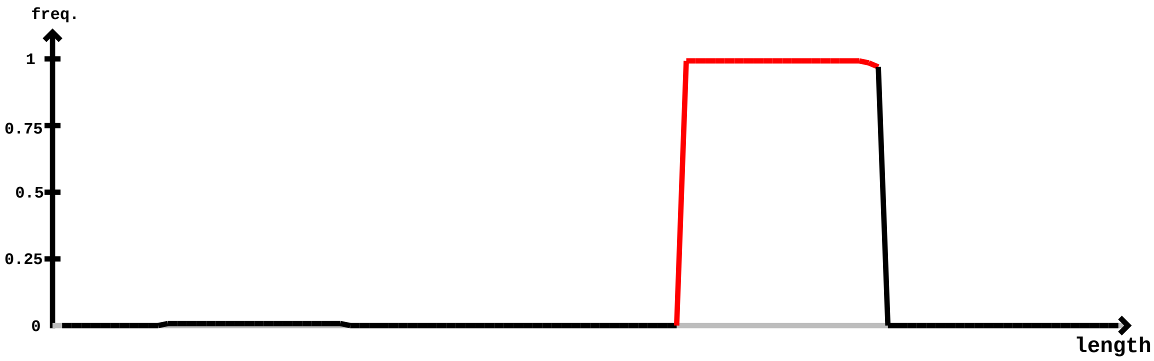

oar-miR-323c

```
5'- gcgugcugcugcacuugaugcuugaggagagguugcccguggccgguucgcauucuucaugucgcacaauacacggucggccucucuucgguaucaaaucccaccuugca   -3'    exp
    ..((((....))))((((((((.(((((((((((.(((((....((.(((((.....))).)).)).))....))))).))))))))))))))))))))............    reads      mm          sample
    .........gcacuugaugcuugaggag..................................................................................    1          0           seq
    ................................................................cacaauacacggucggccu..............................    1          0           seq
    ................................................................cacaauacacggucggccuc.............................    2          0           seq
    ................................................................cacaauacacggucggccucu............................    129        0           seq
```
